# Different effects of granulocyte colony-stimulating factor and erythropoietin on erythropoiesis

**DOI:** 10.1186/s13287-018-0877-2

**Published:** 2018-05-02

**Authors:** Tzu-Lin Chen, Ya-Wen Chiang, Guan-Ling Lin, Hsin-Hou Chang, Te-Sheng Lien, Min-Hua Sheh, Der-Shan Sun

**Affiliations:** 1 Department of Molecular Biology and Human Genetics, Tzu-Chi University, No. 701, Section 3, Zhong-Yang Road, Hualien, 97004 Taiwan, Republic of China; 2 Institute of Medical Sciences, Tzu-Chi University, Hualien, Taiwan

**Keywords:** Granulocyte colony-stimulating factor, Erythropoietin, Erythropoiesis, Reticulocyte, Erythrocytic mobilization

## Abstract

**Background:**

Red blood cells are the most abundant cells in the blood that deliver oxygen to the whole body. Erythropoietin (EPO), a positive regulator of erythropoiesis, is currently the major treatment for chronic anemia. Granulocyte colony-stimulating factor (G-CSF) is a multifunctional cytokine and a well-known regulator of hematopoietic stem cell proliferation, differentiation, and mobilization. The use of EPO in combination with G-CSF has been reported to synergistically improve erythroid responses in a group of patients with myelodysplastic syndromes who did not respond to EPO treatment alone; however, the mechanism remains unclear.

**Methods:**

C57BL/6 J mice injected with G-CSF or EPO were used to compare the erythropoiesis status and the efficiency of erythroid mobilization by flow cytometry.

**Results:**

In this study, we found that G-CSF induced more orthochromatophilic erythroblast production than did EPO in the bone marrow and spleen. In addition, in contrast to EPO treatments, G-CSF treatments enhanced the efficiency of the mobilization of newly synthesized reticulocytes into peripheral blood. Our results demonstrated that the effects of G-CSF on erythropoiesis and erythrocytic mobilization were independent of EPO secretion and, in contrast to EPO, G-CSF promoted progression of erythropoiesis through transition of early stage R2 (basophilic erythroblasts) to late stage R4 (orthochromatophilic erythroblasts).

**Conclusions:**

We demonstrate for the first time that G-CSF treatments induce a faster erythropoiesis-enhancing response than that of EPO. These findings suggest an alternative approach to treating acute anemia, especially when patients are experiencing a clinical emergency in remote areas without proper blood bank supplies.

## Background

Red blood cells (RBCs) are the most abundant cells in the blood and are essential for oxygen transport around the body. After birth, the site of erythropoiesis switches from the fetal liver to the bone marrow (BM) and spleen. In humans, the BM is the major site for steady-state erythropoiesis. In contrast, in mice, in addition to the BM, the spleen plays a minor role (10%) in steady-state erythropoiesis [1, 2]. Under stressful conditions, such as bleeding or acute anemia, the spleen in humans and mice plays a major role in stress erythropoiesis [3, 4]. Hematopoietic stem cells (HSCs) reside in BM niches where cytokines or signals generated by stromal cells consisting of endothelial cells, osteoblasts, and macrophages regulate the differentiation of various blood lineage, including erythroid cells [5]. HSCs first differentiate into megakaryocyte-erythroid progenitors, subsequently into the burst-forming unit-erythroids (BFU-Es), and finally into the colony-forming unit-erythroids (CFU-Es). CFU-Es are more mature than BFU-Es and appear earlier, namely at 2–3 days in mice and 5–8 days in humans, compared with 5–8 days in mice and 10–14 days in humans for BFU-Es. In addition, CFU-Es form smaller colonies than do BFU-Es when cultured in methylcellulose [2, 3]. Erythropoietin (EPO), a 30.4-kDa glycoprotein mainly synthesized by the kidneys, is the main regulator of erythroid cell proliferation, differentiation, and survival [6]. EPO production is upregulated under hypoxic conditions through the activity of the hypoxia-inducible transcription factor (HIF) [7, 8]. The EPO receptor (EPOR) is expressed dominantly on CFU-Es and gradually downregulated during erythroid differentiation [9, 10]. Upon stimulation initiated by EPO binding to the EPORs, CFU-Es develops into proerythroblasts, subsequently into basophilic erythroblasts, then polychromatic erythroblasts, and finally orthrochromatic erythroblasts. The final stage of erythroid differentiation involves the enucleation and maturation of reticulocytes into circulating erythrocytes [11].

Anemia can develop from loss of RBCs, a reduction in RBC production, increased destruction of RBCs, or a shorter RBC lifespan. The World Health Organization defines anemia as a hemoglobin level lower than 12 g/dl in women and lower than 13 g/dl in men [12]. Except for patients with inherited hematopoietic disease, the highest rates of anemia are observed in patients with chronic diseases such as those of the kidney and heart, cancer, inflammatory bowel disease, rheumatoid arthritis, and human immunodeficiency virus (HIV) [13]. Recombinant human EPO (rhEPO) has been used medically for more than 20 years and has generated a multibillion dollar market annually. However, since 1998, some severe adverse effects such as EPO-associated pure red cell aplasia by long-term EPO injection induced neutralizing antibodies have been reported [14, 15]. Other side effects such as hypertension, increased risk of venous thromboembolism, stroke, and death have also been reported [16]. Currently available erythropoiesis-stimulating agents (ESAs) are variations of EPO generated by human cells or Chinese hamster ovary cells. New ESAs (e.g., peptide-based erythropoietic agents), activation of endogenous EPO production through HIF stabilization and GATA1 inhibition, and EPO gene therapy have been developed; however, none of these new methods have shown efficacy superior to that of existing ESAs [17]. Taken together, the development of new anemia therapies with satisfactory levels of efficacy and safety and faster action is required.

Our previous study found that granulocyte colony-stimulating factor (G-CSF) could mobilize newly synthesized erythrocytes to the peripheral blood (PB) and promote erythrocytic differentiation and proliferation in vitro and ex vivo [18]. The present study further investigated the differences between G-CSF and EPO on erythropoiesis. After mice had been treated with G-CSF and EPO, the erythropoiesis status in the BM and spleen were compared using flow cytometry. The stimulation of early erythroid progenitor subsets and temporal regulation of erythroid progenitor mobilization were characterized. The mechanism of G-CSF promotion of erythropoiesis was also discussed.

## Methods

### Toxins and mice

*B. anthracis* lethal toxin (LT) was provided by the Institute of Preventive Medicine, National Defense Medical Center (Taipei, Taiwan), and purified as previously described [19]. EGFP mice [C57BL/6 J-Tg (Pgk1-EGFP) 03Narl] provided by Professor Chou CK [20] and C57BL/6 J mice were purchased from the National Laboratory Animal Center (Taipei, Taiwan). Animals were maintained in a specific pathogen-free environment in the experimental animal center of Tzu Chi University (Hualien, Taiwan).

### Analysis of erythropoiesis status in the BM and spleen

C57BL/6 J mice (male, 10–12 weeks old) were retro-orbitally injected with 2 IU/g rhEPO (Neorecormon®, Roche, Mannheim, Germany) or 55 μg/kg recombinant human G-CSF (Filgrastim, Kirin, Tokyo) once daily on 3 consecutive days. Animals treated with an identical volume of saline served as negative controls. BM cells were isolated as previously described [21]. Spleens were minced using the plunger of a 50-ml syringe and resuspended using a P1000 Pipetman (Gilson, Middleton, WI, USA) to create a single-cell suspension. Cells were blocked with RPMI-1640 medium (Gibco Laboratories) containing 5% bovine serum albumin at 37 °C for 1 h and subsequently incubated with rat anti-mouse fluorescein isothiocyanate-conjugated CD71 antibody (BioLegend) and rat antimouse allophycocyanin (APC)-conjugated TER-119 antibody (BD Immunocytometry System) at 37 °C for 1 h. After washing with phosphate-buffered saline (PBS), the cells were resuspended in 1 ml of PBS and analyzed using a Beckman Coulter Gallios™ flow cytometer (Beckman Coulter, CA, USA).

### Flow cytometry analysis of mobilized erythrocytes in EGFP mice

Enhanced green fluorescent protein (EGFP) mice (male, 8 months old) were injected with 55 μg/kg G-CSF or 2 IU/g EPO retro-orbitally once daily on 3 consecutive days. PB was collected retro-orbitally at 20, 40, and 60 h after initial injection. Cells were incubated with rat anti-mouse APC-conjugated TER-119 antibody at 37 °C for 1 h and subsequently with 5 μM of RNA-selective dye F22 [22] at 37 °C for 30 min. After washing with PBS, the cells were analyzed using a Beckman Coulter Gallios™ flow cytometer.

### EPO and soluble P-selectin immunoassay

C57BL/6 J mice (male, 10–12 weeks old) were treated with 55 μg/kg G-CSF administered by retro-orbital injection once daily on 2 consecutive days for the EPO immunoassay or 5 consecutive days for the soluble P-selectin immunoassay. Animals treated with an identical volume of saline served as negative controls. C57BL/6 J mice that were retro-orbitally injected with 1.5 mg/kg LT and untreated served as positive controls for the EPO immunoassay and the soluble P-selectin immunoassay, respectively. PB (serum for the EPO immunoassay and plasma for the soluble P-selectin immunoassay) was collected retro-orbitally at 22, 44, and 66 h after initial injection for the EPO immunoassay, and day 0 before initial injection and days 1–5 after initial injection for the soluble P-selectin immunoassay. The EPO and soluble P-selectin immunoassays were performed using an enzyme-linked immunosorbent assay (ELISA) in accordance with the manufacturers’ instructions (Quantikine® Mouse/Rat EPO immunoassay, R&D systems; Mouse P-selectin ELISA kit, RayBiotech, Inc., USA).

### Hematopoietic parameter detection

C57BL/6 J mice (male, 10–12 weeks old) were treated with 55 μg/kg G-CSF or 1 mg/kg recombinant mouse P-selectin (purified mouse P-selectin–IgG fusion protein; BD Pharmingen) once daily on 2 consecutive days through retro-orbital injection. The hematopoietic parameters were measured on day 2 after initial injection using an automated hematology analyzer (XP-300, Sysmex Corporation).

### Statistics

All quantifiable data are presented as mean ± standard deviation (SD). Statistical analysis was conducted by one-way analysis of variance (ANOVA) followed by the post-hoc Bonferroni-corrected *t* test using the SPSS software, version 17.0 (SPSS Inc., Chicago, IL, USA). *P* values lower than 0.05 indicated significant differences.

## Results

### G-CSF enhanced R4 erythroblast cell production to a greater extent than EPO did in the BM and spleen

**Fig. 1:**
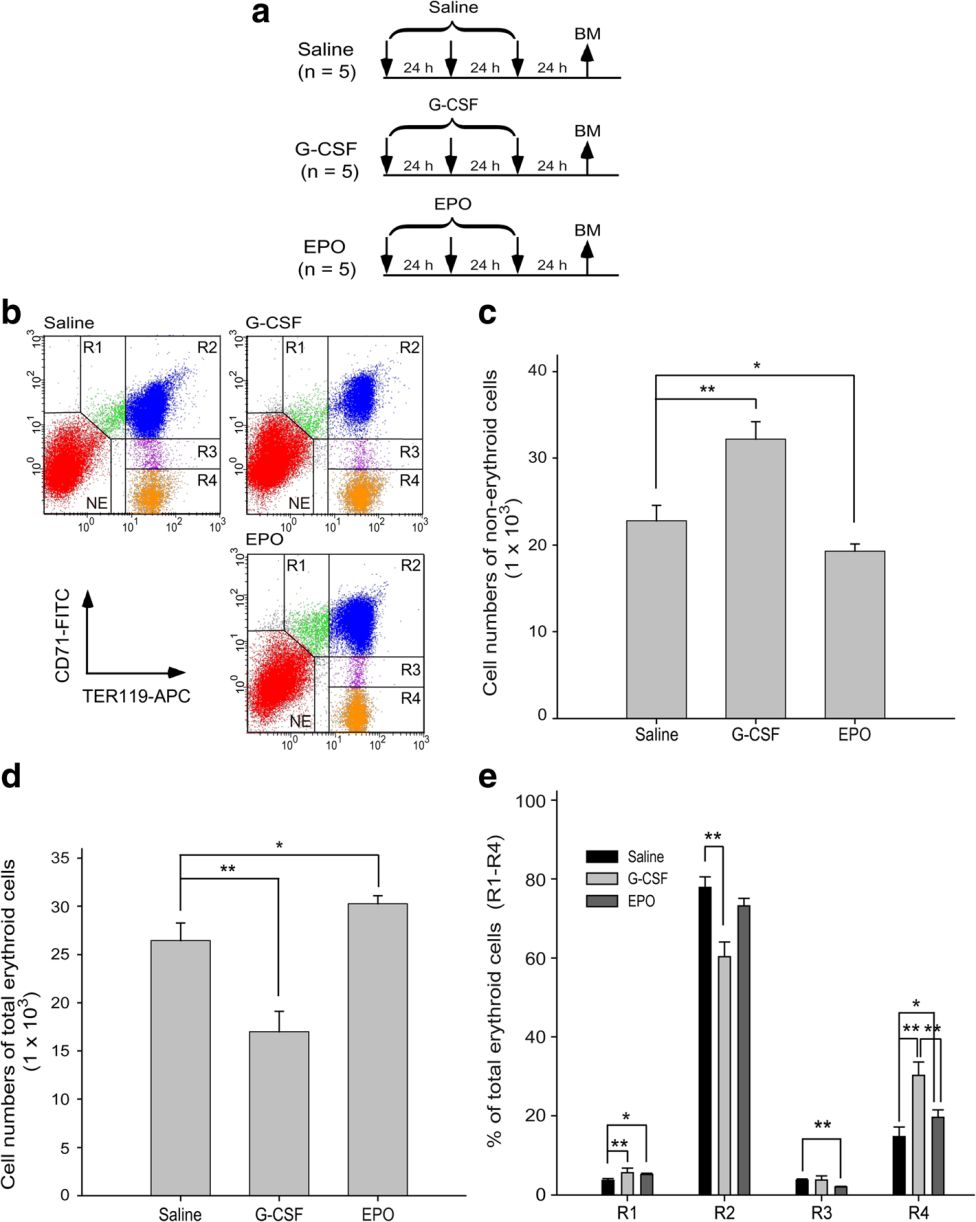
Granulocyte colony-stimulating factor (G-CSF) treatment induced more R4 erythroblast cells than erythropoietin (EPO) treatment did in bone marrow (BM). **a** The experimental outline used to measure erythropoiesis status after G-CSF (*n* = 5) and EPO (*n* = 5) treatments in the BM is depicted. Saline-treated mice (*n* = 5) were used as negative controls. Flow cytometry analysis was performed 72 h after initial injection. The erythroblast cells were gated as R1 (CD71^high^, TER-119^med^), R2 (CD71^high^, TER-119^high^), R3 (CD71^med^, TER-119^high^), and R4 (CD71^low or –^, TER-119^high^) in all groups. Nonerythroid cells were gated as NE (TER-119 negative) (**b**). Cell numbers of nonerythroid (**c**) and total erythroid cells (sum of R1, R2, R3, and R4) (**d**), and the percentage of individual erythroblasts (R1, R2, R3, and R4) in the total number of erythroid cells in each group (**e**) were quantified. Data are reported as mean ± SD. *P < 0.05, **P < 0.01

**Fig. 2:**
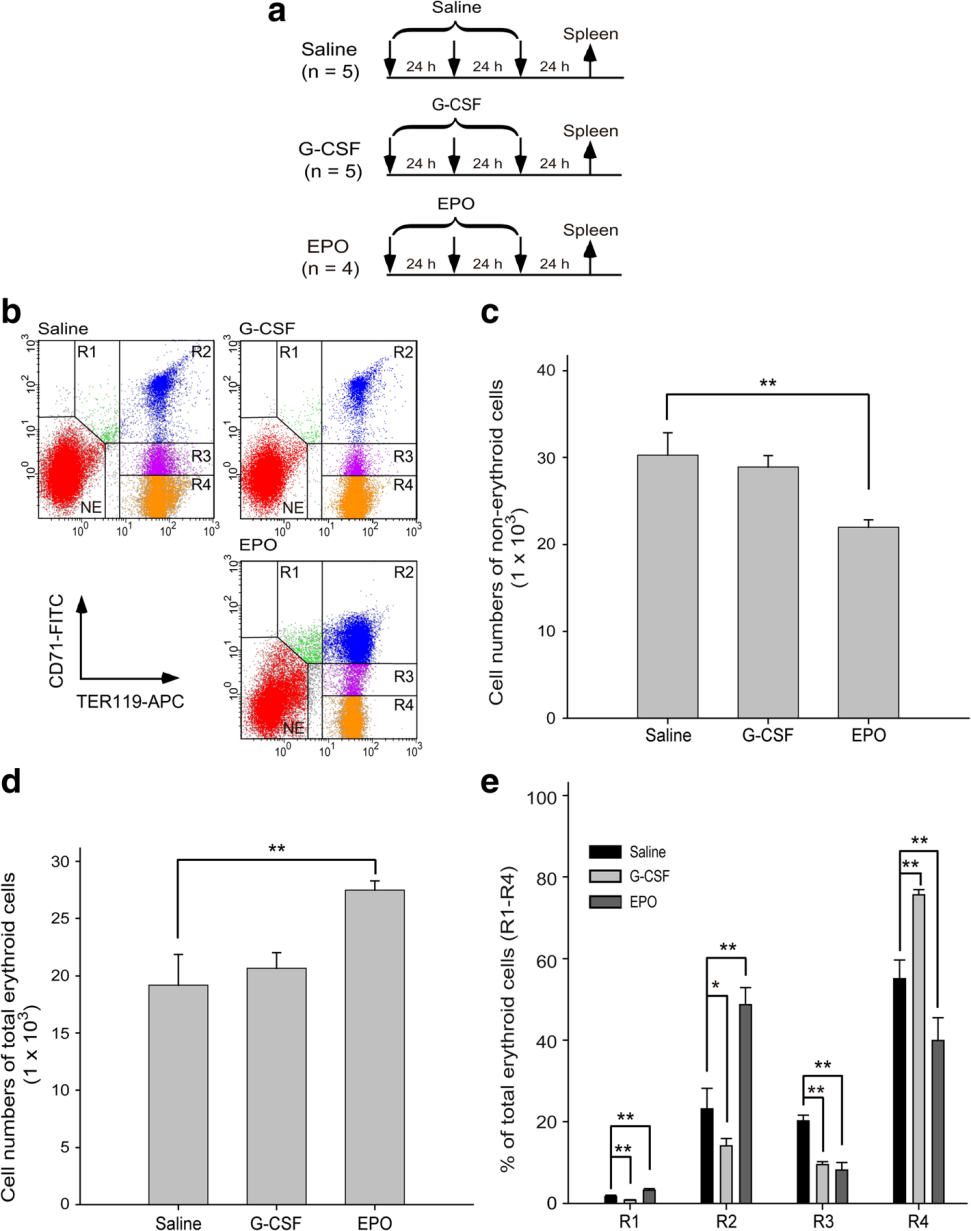
Granulocyte colony-stimulating factor (G-CSF) treatment induced more R4 erythroblast cells than erythropoietin (EPO) treatment did in the spleen. **a** The experimental outline used to measure the erythropoiesis status after G-CSF (*n* = 5) and EPO (*n* = 4) treatments in the spleen is depicted. Saline-treated mice (*n* = 5) were used as negative controls. Flow cytometry analysis was performed 72 h after initial injection. The erythroblast cells were gated as R1, R2, R3, and R4 in all groups (see Fig. 1 for details). Nonerythroid cells were gated as NE (**b**). Cell numbers of nonerythroid (**c**) and total erythroid cells (sum of R1, R2, R3, and R4) (**d**), and the percentage of individual erythroblasts (R1, R2, R3, and R4) in the total number of erythroid cells in each group (**e**) were quantified. Data are reported as mean ± SD. *P < 0.05, **P < 0.01

To compare the erythropoiesis status after G-CSF and EPO treatments, C57BL/6 J mice were retro-orbitally injected with G-CSF or EPO once daily for 3 consecutive days. Erythropoiesis progression was analyzed in the BM (Fig. 1a) and spleen (Fig. 2a) through flow cytometry. Using antibodies against the erythroid markers CD71 and TER119, erythroblasts were divided into the following four populations from early to late sequential differentiation stages: proerythroblasts (R1, CD71^high^/TER-119^med^), basophilic erythroblasts (R2, CD71^high^/TER-119^high^), late basophilic and polychromatophilic erythroblasts (R3, CD71^med^/TER-119^high^), and orthochromatophilic erythroblasts (R4, CD71^low or −^/TER-119^high^) (Figs. 1b and 2b) [18, 21, 23]. In agreement with the findings of other studies, in mouse BM, G-CSF enhanced more nonerythroid cell populations (NE, TER-119^−^) [24] than EPO did (Fig. 1c). In contrast, EPO substantially increased the total number of erythroid cells [6] (Fig. 1d). Although total erythroid cell counts in the BM of mice from the G-CSF-treated groups were lower than those of mice from the EPO-treated groups, G-CSF elicited a markedly larger R4 population than EPO did (Fig. 1e).

The spleen is a secondary organ for erythropoiesis but not for granulopoiesis [2–4, 25]. In characterizing the differentiation progression in the mouse spleen, we found that G-CSF treatments did not increase the nonerythroid cell population or total erythroid cell number (Fig. 2c, d). Compared with the responses in the BM, EPO upregulated the percentage of early erythroblasts (R1 and R2) and downregulated the late stages of erythroblasts (R3 and R4) (Fig. 2e), whereas G-CSF reduced the percentage of early R1, R2, and R3 cells and considerably increased the number of late-stage R4 erythroblasts (Fig. 2e). These results collectively suggest that G-CSF enhances late-stage R4 erythroblast cell production to a greater extent than EPO does in the BM and spleens of mice.

### G-CSF mobilized more newly synthesized reticulocytes than EPO did

**Fig. 3:**
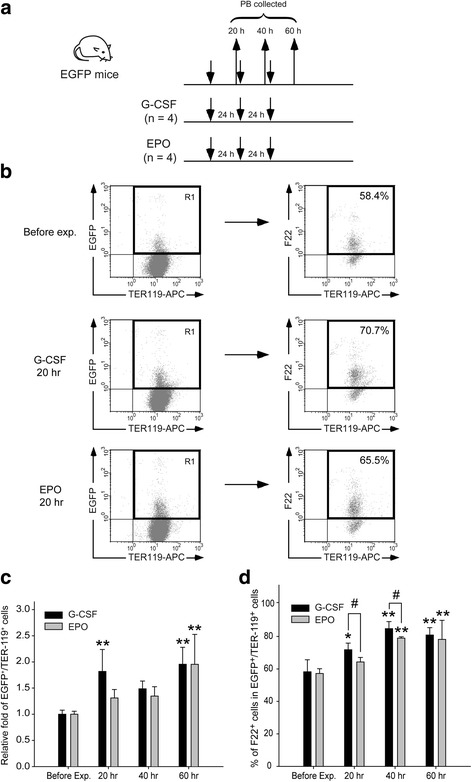
Granulocyte colony-stimulating factor (G-CSF) treatment mobilized more newly synthesized reticulocytes than erythropoietin (EPO) treatment did. **a** The experimental outline for investigating erythrocyte mobilization in enhanced green fluorescent protein (EGFP) mice after G-CSF (*n* = 4) and EPO (*n* = 4) treatments is shown. Peripheral blood (PB) was collected 20, 40, and 60 h after initial injection. Flow cytometry analysis was performed to analyze the relative fold of EGFP^+^/TER-119^+^ cells (gated R1 region) compared with the before experiment (Before exp.) groups and the percentage of F22^+^ cells in EGFP^+^/TER-119^+^ cells at 20 h (**b**). Quantitative results of the relative fold of EGFP^+^/TER-119^+^ cells (**c**) and the percentage of F22^+^ cells in EGFP^+^/TER-119^+^ cells at 20, 40, and 60 h (**d**) are indicated. Data are reported as mean ± SD. *P < 0.05, **P < 0.01, versus the Before exp. groups; ^#^*P* < 0.05

Using an EGFP transgenic mouse model, we found that G-CSF caused newly synthesized erythrocytes to mobilize to the PB and induced erythrocytes to mobilize into the PB faster than EPO [18]. After using the same experimental strategy (Fig. 3a), our data revealed that the mobilization efficiencies of newly synthesized erythrocytes (R1, EGFP^high^/TER-119^high^) were higher at 20 h under G-CSF treatment than under EPO, and were similar between G-CSF and EPO treatments at 40 and 60 h after initial injection (Fig. 3b, c). Because G-CSF promoted more R4 erythroblast synthesis than EPO did in the BM and spleen (Figs. 1 and 2), we hypothesized that G-CSF may promote reticulocyte mobilization to a greater extent than EPO. Because only reticulocytes exhibit residue ribonucleic acid (RNA) expression among newly synthesized erythrocyte populations [26], F22, a RNA-selective dye [22], combined with TER-119 was used to discriminate reticulocytes from matured erythrocytes in newly synthesized erythrocytes. Over two time courses, 20 and 40 h, G-CSF preferred to mobilize newly synthesized reticulocytes (F22^high^/EGFP^high^/TER-119^high^) to a greater extent than EPO did (Fig. 3b, d).

### G-CSF-dependent enhancement of erythropoiesis and RBC mobilization were not mediated by eliciting EPO and the P-selectin pathway

**Fig. 4:**
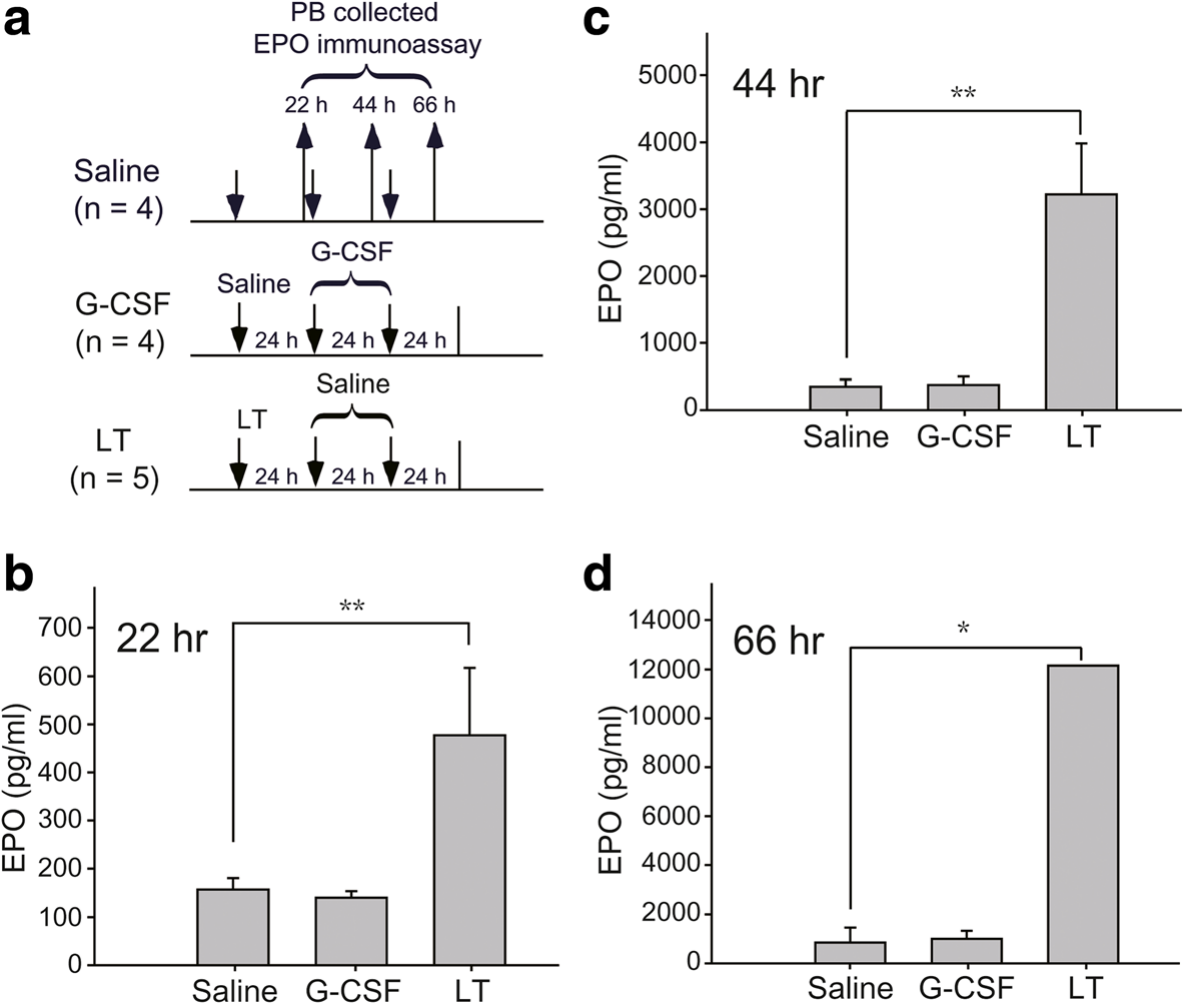
Granulocyte colony-stimulating factor (G-CSF) treatment did not trigger erythropoietin (EPO) secretion. **a** The experimental outline used for the EPO immunoassay is illustrated. Mice were injected with G-CSF (*n* = 4) once daily after saline injection. EPO concentrations in the peripheral blood (PB) were detected by ELISA at 22 h (**b**), 44 h (**c**), and 66 h (**d**) after initial injection. Saline (*n* = 4) and anthrax lethal toxin (LT) (*n* = 5) treatments served as negative and positive controls, respectively. Data are reported as mean ± SD. *P < 0.05, **P < 0.01

The transcription factor HIF-1α can be upregulated by G-CSF and then binds to an EPO promoter to increase circulating EPO levels after five consecutive G-CSF injections [27]. Accordingly, we hypothesized that G-CSF may stimulate erythropoiesis and mobilize erythrocytes by increasing the synthesis of EPO. Our experimental design is shown in Fig. 4a. Treatment with *Bacillus anthracis*, a LT that can induce hypoxia-elicited EPO secretion in mice [28], was used as a positive control. In agreement with a previous study [28], our results revealed that circulating EPO levels gradually increased following LT administration in mice [28]. In contrast, circulating EPO levels were not upregulated at all examined time points after G-CSF treatment in mice (Fig. 4b–d). These results suggest that the G-CSF-dependent enhancement of erythropoiesis and RBC mobilization are not mediated by eliciting EPO.

**Fig. 5:**
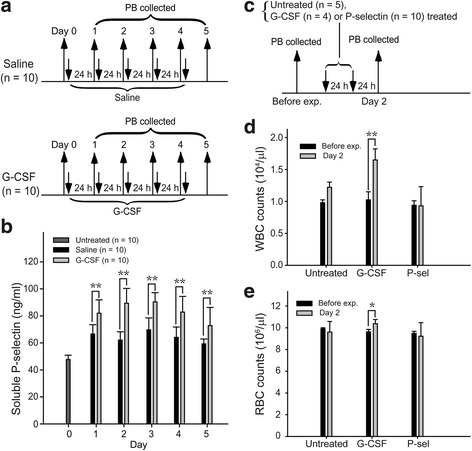
Secretion of soluble P-selectin (P-sel) after granulocyte colony-stimulating factor (G-CSF) treatment was independent of erythrocytic mobilization. **a** The experimental outline to monitor the concentrations of soluble P-selectin after G-CSF treatment (*n* = 10) is illustrated. Untreated (*n* = 10) and saline-treated groups (*n* = 10) served as negative controls. The concentrations of soluble P-selectin were detected by ELISA (**b**). The experimental outline to detect the cell counts of white blood cells (WBCs) (**d**) and red blood cells (RBCs) (**e**) in PB after G-CSF (*n* = 4) and P-selectin (*n* = 10) treatments is shown (**c**). Untreated groups (*n* = 5) served as negative controls. Data are reported as mean ±± SD. *P < 0.05, **P < 0.01

P-selectin is widely recognized as a cell adhesion receptor that mediates leukocyte rolling through binding to glycosylated moieties of the main ligand P-selectin glycoprotein ligand 1 (PSGL-1) [29]. The same property of P-selectin enables HSCs to adhere to stromal cells, especially vesicular endothelial cells, through P-selectin (HSC)–PSGL-1 (stroma) interactions [30]. G-CSF induced greater and faster mobilization of myeloid cells from BM in PSGL-1 knockout mice than in wild-type mice [31]. Studies have also suggested that G-CSF administration increases levels of circulating soluble P-selectin [32, 33]. This evidence collectively suggests that P-selectin/PSGL-1-mediated binding between erythroid precursors and stromal cells may partially control the mobilization and release of progenitor cells from BM into the blood stream, thereby indicating the possibility that G-CSF may enhance erythrocytic mobilization by eliciting circulating soluble P-selectin to compete with the P-selectin/PSGL-1-mediated precursor–stroma interaction. To reproduce G-CSF-mediated elicitation of circulating soluble P-selectin, G-CSF was injected into C57BL/6 J mice on 5 consecutive days (Fig. 5a). The levels of circulating soluble P-selectin increased significantly after G-CSF injection (Fig. 5b). Next, we designed an experiment to examine whether soluble P-selectin can increase the RBC counts in the PB (Fig. 5c). Although two doses of G-CSF increased the white blood cell (WBC) and RBC counts in the PB, the same effect was not observed when two equivalent doses of soluble P-selectin were applied (Fig. 5d, e). This finding suggests that G-CSF mobilization of erythrocytes into the PB is not primarily mediated through the P-selectin pathway.

## Discussion

This study demonstrated that stimulation of erythropoiesis in mice with G-CSF is different to that with EPO. Treatment with G-CSF elicited greater R4 erythroblast production than did treatment with EPO in the BM and spleen. Additionally, G-CSF induced higher mobilization levels of newly synthesized reticulocytes from the BM and spleen to the PB than EPO did.

G-CSF has been a US Food and Drug Administration (FDA)-approved drug for treating various hematopoietic defects for many years. In contrast to EPO, elicitation of neutralizing antibodies after G-CSF treatments has not been reported. Myelodysplastic syndromes (MDS) are a diverse group of diseases characterized by ineffective hematopoiesis and peripheral cytopenia with unknown mechanisms [34, 35]. As one of the disease hallmarks, approximately 85% of patients with MDS manifest anemia [36, 37]. Synergism between G-CSF and EPO treatments has ameliorated anemic conditions in groups of patients with MDS who did not respond to EPO treatments [38, 39]. This suggests that the erythropoiesis-enhancing effect of the combination of G-CSF and EPO has been empirically recognized by physicians and the medical community. Studies have shown that G-CSF has antiapoptotic effects that protect erythroblasts from cell death [40–42]. However, the effects and mechanisms of G-CSF on erythropoiesis in vivo must be elucidated. Our results demonstrate that G-CSF could promote erythropoiesis by stimulating R4 erythroblast production and then mobilizing newly synthesized reticulocytes to a greater extent than EPO treatment. The effects partially agree with the findings of our previous study that small and medium progenitor cell colonies appeared earlier when tested with an erythroid colony-forming cell assay [18]. In contrast, treatments with G-CSF alone do not exhibit ameliorative effects on anemia in patients with MDS [42, 43]. In accordance with these findings, in our erythroid colony-forming cell experiment, G-CSF exhibited erythropoietic properties only when used with EPO [18]. This finding implied that G-CSF was unable to stimulate erythropoiesis without EPO. The EPOR is expressed dominantly on CFU-Es and gradually downregulated during erythroid differentiation [9, 10]. In addition, the G-CSF receptor is expressed on erythroid progenitors [42]. Because late-stage erythroblasts (R3 or R4) were upregulated after G-CSF treatment, synergism between G-CSF and EPO is likely accomplished by accelerating the erythropoietic process into the late stage. Recently, papers reported that G-CSF treatments enhanced hematopoietic stem and progenitor cell mobilization by enhancement of dipeptidylpeptidase 4 (CD26) activities and vascular permeability in the BM [44, 45]. This evidence may provide an additional explanation as to why G-CSF induced higher mobilization levels of newly synthesized reticulocytes from the BM and spleen to the PB than that of EPO. Although the responses that occur in BM with multilineage cell interactions are difficult to analyze, the mechanism warrants further investigation.

## Conclusions

In conclusion, although G-CSF combined with EPO has been administered to treat anemia in patients with MDS, aplastic anemia, and HIV for many years [38, 39, 46, 47], the mechanism through which G-CSF promotes erythropoiesis remains unclear. This study demonstrates that G-CSF promotes erythropoiesis independent of the secretion of EPO and soluble P-selectin. In addition, G-CSF induces the production of levels of R4 erythroblasts to a greater extent than EPO does in the BM and spleen, and treatment with G-CSF mobilizes more newly synthesized reticulocytes to the PB than EPO does. These findings indicate an alternative method for ameliorating anemia, especially in situations where a patient is in immediate need of oxygen, such as those involving infectious diseases [18] or with clinical urgency in remote areas.

## Abbreviations

APC: Allophycocyanin
BFU-E: Burst-forming unit-erythroid
BM: Bone marrow
CFU-E: Colony-forming unit-erythroid
EGFP: Enhanced green fluorescent protein
ELISA: Enzyme-linked immunosorbent assay
EPO: Erythropoietin
EPOR: Erythropoietin receptor
ESA: Erythropoiesis-stimulating agent
FDA: Food and Drug Administration
G-CSF: Granulocyte colony-stimulating factor
HIF: Hypoxia-inducible transcription factor
HIV: Human immunodeficiency virus
HSC: Hematopoietic stem cell
LT: lethal toxin
MDS: Myelodysplastic syndromes
PB: Peripheral blood
PBS: Phosphate-buffered saline
PSGL-1: P-selectin glycoprotein ligand 1
RBC: Red blood cell
rhEPO: Recombinant human erythropoietin
RNA: Ribonucleic acid
SD: Standard deviation
